# Extended Deletion in the *GBA1* Gene, Masked by Apparent p.Asn409Ser Homozygous: Diagnostic Implications and the Need for an Integrated Genetic Approach

**DOI:** 10.3390/ijms27209123

**Published:** 2026-10-15

**Authors:** Monia Anania, Miriam Giacomarra, Giovanna Palumbo, Annalisa D’Errico, Emanuela Maria Marsana, Silvia Palladino, Giorgio Sforzini, Daniele Francofonte, Paolo Colomba, Giovanni Duro, Carmela Zizzo

**Affiliations:** 1 Institute for Biomedical Research and Innovation (IRIB), National Research Council (CNR), 90146 Palermo, Italy; 2 Department of Translational and Precision Medicine, “La Sapienza” University of Rome, 00161 Rome, Italy

**Keywords:** Gaucher disease, glucocerebrosidase, Multiplex Ligation–dependent Probe Amplification (MLPA), *GBA1*

## Abstract

This case report describes a 22-year-old woman with a phenotype compatible with Gaucher disease, characterized by hepatomegaly, splenomegaly, and thrombocytopenia. β-glucocerebrosidase activity was lower than reference values, making further genetic testing necessary. Sanger sequencing of the *GBA1* gene revealed the presence of the pathogenic variant p.N409S in apparent homozygosity. Plasma glucosylsphingosine (Lyso-Gb1) testing provided further biochemical support for the diagnosis. Family screening, by Sanger sequencing, showed that the father is a heterozygous carrier of the p.N409S variant, while the mother and brother do not have any variants in the *GBA1* gene. The absence of the maternal variant suggested the possible presence, in the proband, of a second structural alteration that could not be detected by traditional Sanger sequencing. As a result, an analysis was performed using Multiplex Ligation–dependent Probe Amplification (MLPA), which documented a large heterozygous deletion involving exon 6 in both the proband and her mother, allowing us to define a mutational pattern consisting of p.Asn409Ser in trans with an exon deletion. This case report demonstrates the importance of an integrated diagnostic approach in the analysis of genetic diseases, in which clinical evaluation, biochemical tests and molecular techniques complement each other. The combination of family studies and methods complementary to Sanger sequencing, such as analyses for the detection of structural rearrangements, is essential for accurate genotypic characterization. This approach is essential not only for the correct interpretation of the genetic pattern, but also for providing appropriate genetic counselling and defining optimal clinical management of the patient.

## 1. Introduction

Gaucher disease (GD; OMIM #230800, ORPHA355) is a rare autosomal recessive genetic disorder caused by pathogenic variants in the *GBA1* gene, located on chromosome 1q21. This gene encodes the lysosomal enzyme acid β-glucosidase (glucocerebrosidase; EC 3.2.1.45), which is responsible for the hydrolysis of glucosylceramide into ceramide and glucose [1,2,3,4]. Reduced enzyme activity causes glucosylceramide to accumulate inside macrophages, which transform into the characteristic Gaucher cells. These cells can infiltrate the bone marrow, liver, spleen and other areas, causing the multisystemic clinical picture typical of the disease [5].

Although GD is traditionally classified into three main forms, the clinical spectrum is broad and continuous. The phenotypic presentation is extremely heterogeneous and can range from mild to severely progressive forms, with variable neurological involvement. This variability complicates differential diagnosis and can lead to significant diagnostic delays, postponing access to enzyme replacement therapy (ERT) or substrate reduction therapy (SRT), which are essential for preventing irreversible complications [6].

The diagnostic suspicion is based on the integration of clinical evaluation, laboratory tests and family history. The diagnosis is traditionally confirmed by measuring glucocerebrosidase activity, often performed on dried blood spots (DBS), which is absent or reduced in affected patients [7]. However, measuring enzyme activity alone does not allow for a complete characterization of the disease. The introduction of specific biomarkers, in particular glucosylsphingosine (Lyso-Gb1), has significantly improved diagnostic accuracy, allowing for better definition of clinical severity and therapeutic monitoring [8,9,10,11].

Genetic analysis of the *GBA1* gene is essential for defining genotype, genetic counselling and prognostic assessment. However, given the autosomal recessive nature of GD and the presence of structural variants that cannot be detected by traditional sequencing, it should be considered complementary to enzymatic analysis, which remains the primary diagnostic criterion.

In this case report, we describe the case of a 22-year-old woman with a clinical picture consistent with GD, initially classified as homozygous for the p.N409S variant (N370S). Further investigations identified a composite genotype p.Asn409Ser/del exon 6. This case highlights the complexity of genetic analysis of the *GBA1* gene and emphasizes the need for an integrated diagnostic approach combining clinical expertise, specific biomarkers and complementary molecular techniques, including methods for detecting deletions and family studies.

## 2. Case Presentation

A 22-year-old female patient was evaluated in the haematology department following the presentation of a clinical picture characterized by hepatomegaly, splenomegaly and thrombocytopenia. The exclusion of other possible diagnoses and the clinical suspicion of Gaucher disease led to specific molecular tests for this condition undergoing investigation. Analysis of lysosomal glucocerebrosidase enzyme activity, conducted on whole blood samples dried on filter paper (Dried Blood Filter Paper Test), according to the methodology described by Chamoles et al. [12], with modifications, showed a value of 0.5 nmol/h/mL, significantly lower than the normal reference range (≥2.5 nmol/h/mL) (Table 1). This finding made it essential to confirm the diagnosis through genetic analysis and determination of the glucosylsphingosine LysoGB1 biomarker.

**Figure 1:**
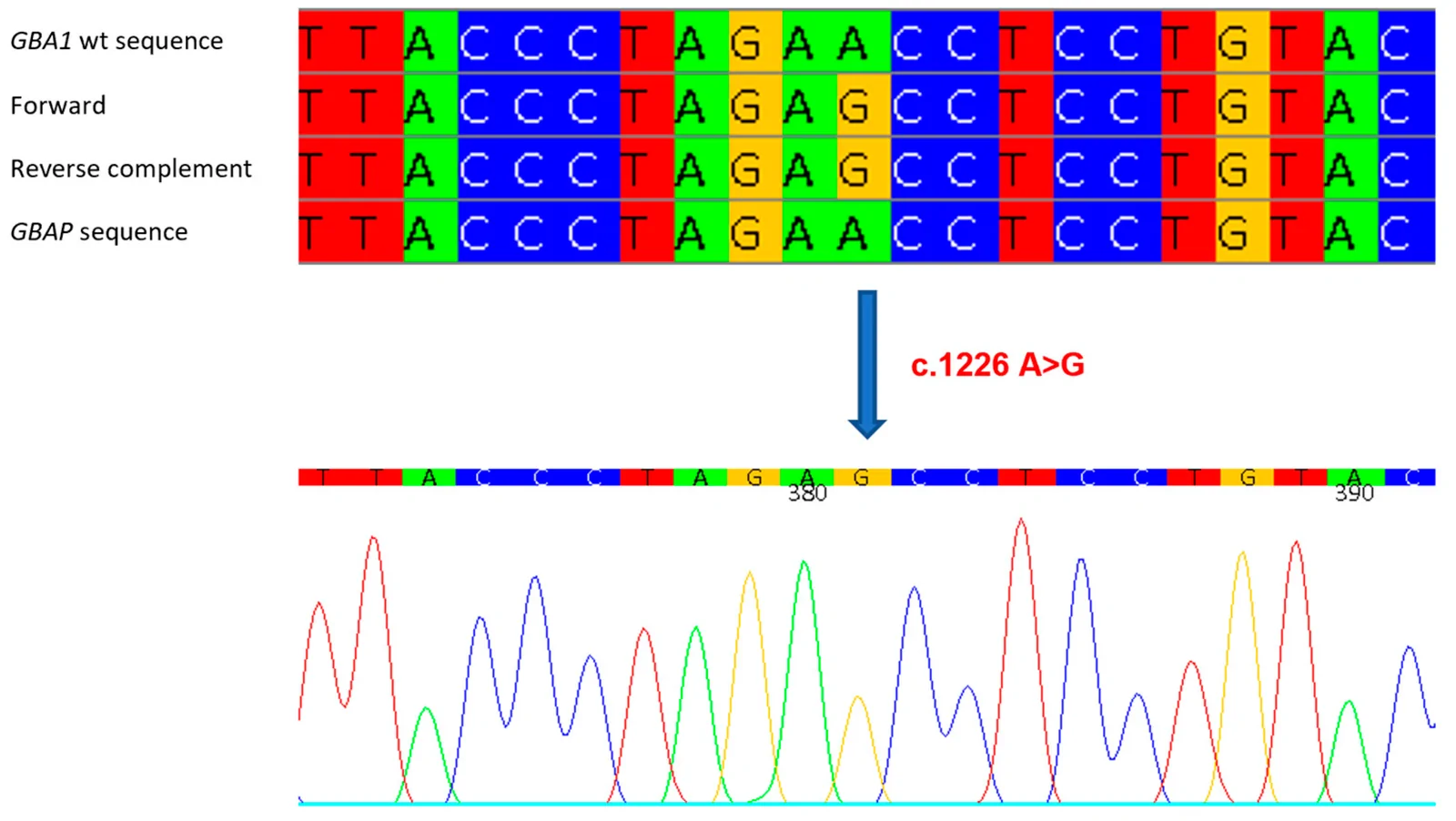
Sanger sequencing shows the c.1226 A>G mutation in homozygosity in the proband. Each base corresponds to a color: red for Thymine, blue for Cytosine, yellow for Guanine and green for Adenine. The arrows indicate the mutation.

Genomic DNA was extracted from dried blood samples on filter paper using the Qiagen EZ1 Advanced XL automatic extractor, in combination with the EZ1 Advanced XL DNA Investigator card, programmed with specific protocols for DNA extraction from filter paper, and the EZ1 DNA Investigator kit. DNA concentrations were quantified using a biophotometer (Eppendorf, Hamburg, Germany). The investigation of mutations in the *GBA-1* gene was conducted using PCR followed by Sanger sequencing. Two pairs of specific PCR primers were designed to analyze the *GBA-1* gene exclusively and identify possible recombinant alleles with the *GBAP* pseudogene. The forward primer for the first amplicon is located 329 bp upstream of the ATG of exon 1 (GBA_EX1FOR-PC 5′-CTCCATGCAAATCTGTGTTC-3′). The reverse primer for the same amplicon is located 6 bases downstream of the end of exon 5 (GBA_EX5Rev-PC 5′-GGCCTGAAAAAGCTAGAATG-3′). The total size of this amplicon is 2952 bp. The forward primer for the second amplicon is positioned 377 bases upstream of exon 5 (GBA_EX5FOR-PC 5′-CCAGGATGATTGCGAACTC-3′). The reverse primer for the same amplicon is positioned 75 bases downstream of the UGA in the exon (11GBA_EX11Rev-PC 5′ TGCTGTGCCCTCTTTAGTC-3′). The total size of this amplicon is 4127 bp [13]. PCR products related to exons 1-intron 5 and intron 5-exon 11 were purified using ExoSAP-ITTM PCR and used for targeted sequencing of exons 1–5 and 5–11 of the *GBA-1* gene. Sanger sequencing was performed by Eurofins Genomics, while sequence analysis was carried out using LI-COR Align IR imaging system (LI-COR Biosciences, Lincoln, NE, USA) and Chromas version 2.5 bioinformatics software. Comparative analysis of the patient’s *GBA-1* gene sequences with the wild-type reference sequence and the *GBAP* pseudogene revealed the presence of a homozygous point mutation at nucleotide c.1226 A>G in exon 9, corresponding to the p.N409S mutation, known in the literature as N370S [14] (Figure 1).

**Figure 2:**
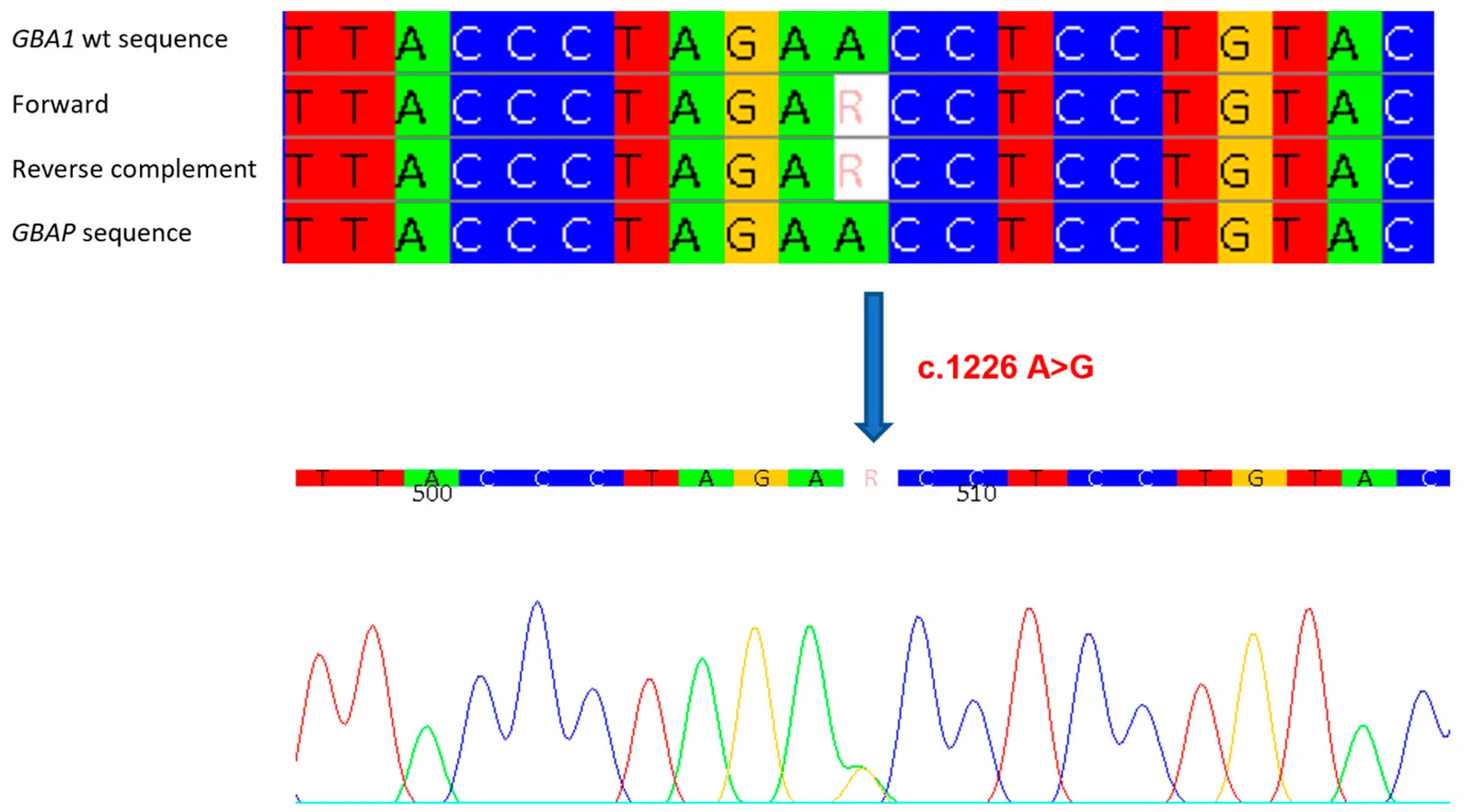
Sanger sequencing shows the c.1226 A>G mutation in heterozygous in the proband’s father. Each base corresponds to a color: red for Thymine, blue for Cytosine, yellow for Guanine and green for Adenine. The arrows indicate the mutation.

Furthermore, quantification of the accumulated metabolite Lyso-Gb1, performed on dried blood samples using the LC-MS/MS technique [15,16,17], showed a value of 498 nmol/L, higher than the standard reference values (normal range ≤ 6.8 ng/mL) (Table 1), thus confirming the diagnosis of Gaucher disease in the patient in question. Genetic analysis was extended to the proband’s immediate family members (mother, father and brother), who did not show any clinical signs compatible with Gaucher disease. Sequencing using the Sanger method detected the presence of the p.Asn409Ser mutation in heterozygosity in the proband’s father (Figure 2), who had an enzyme activity of 4.2 nmol/h/mL (normal range ≥ 2.5 nmol/h/mL) (Table 1) and a Lyso-Gb1 level of 6.8 ng/mL (normal range ≤ 6.8 ng/mL), both of which were within normal reference values; in contrast, no mutations associated with the disease were identified in either the mother or the brother.

**Figure 3:**
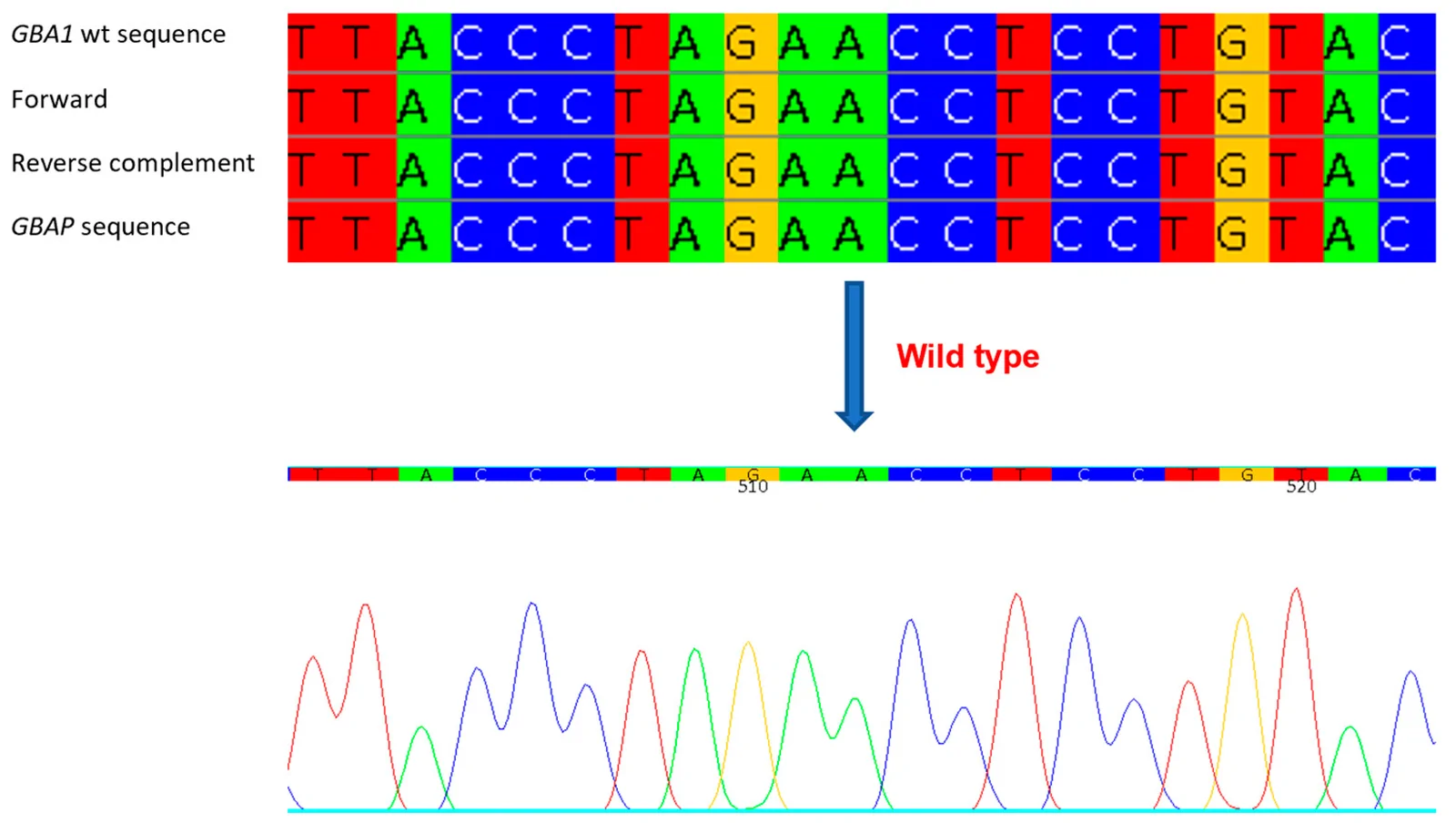
Sanger sequencing shows the wild-type sequence in the proband’s mother. Each base corresponds to a color: red for Thymine, blue for Cytosine, yellow for Guanine and green for Adenine. The arrows indicate the absence of mutation.

**Figure 4:**
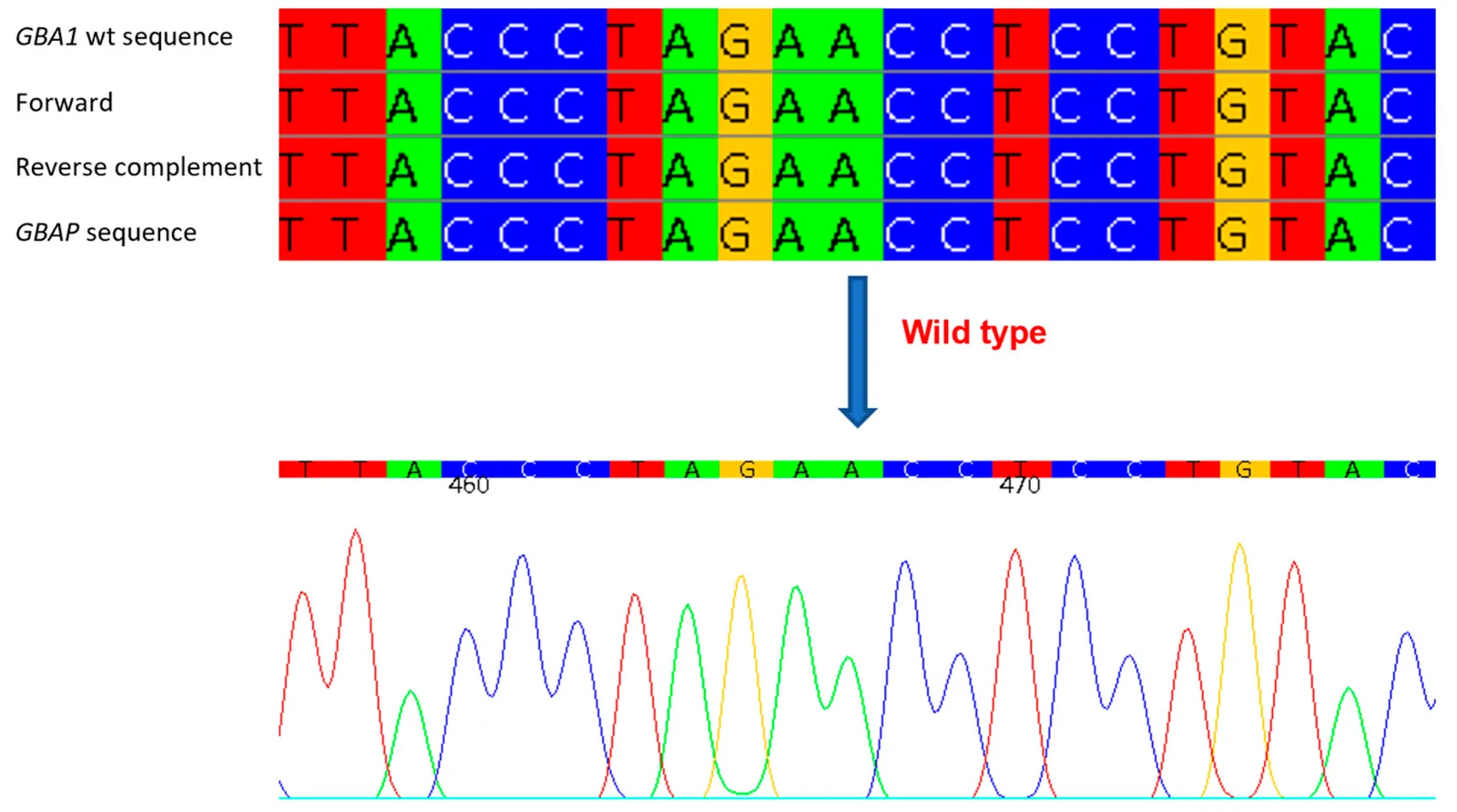
Sanger sequencing shows the wild-type sequence in the proband’s brother. Each base corresponds to a color: red for Thymine, blue for Cytosine, yellow for Guanine and green for Adenine. The arrows indicate the absence of mutation.

The absence of mutations in the brother was consistent with the autosomal recessive nature of Gaucher disease, as both parents could have transmitted both wild-type alleles to their son; however, the absence of this mutation in the mother was inconsistent with the genetic profile of the proband, who was homozygous for the p.Asn409Ser mutation (Figure 3 and Figure 4). Furthermore, both the mother and brother of the proband have enzyme levels and Lyso-Gb1 values within the normal range (Table 1).

The results from the family analysis, using Sanger sequencing, therefore required further diagnostic investigation of both the proband and family members, using the MLPA (Multiplex Ligation Probe Amplification) technique, which allows large deletions or gene insertions to be identified [18]. In particular, the hypothesis of a possible large deletion in heterozygosity on one allele of the proband, not detectable by Sanger sequencing, was formulated in consideration of the absence of mutations in the mother, a situation that could lead to the amplification and sequencing of only one allele, while the other would be entirely or partially deleted. This hypothesis is further supported by the presence of the homozygous p.Asn409Ser mutation in the proband, in Sanger.

**Figure 5:**
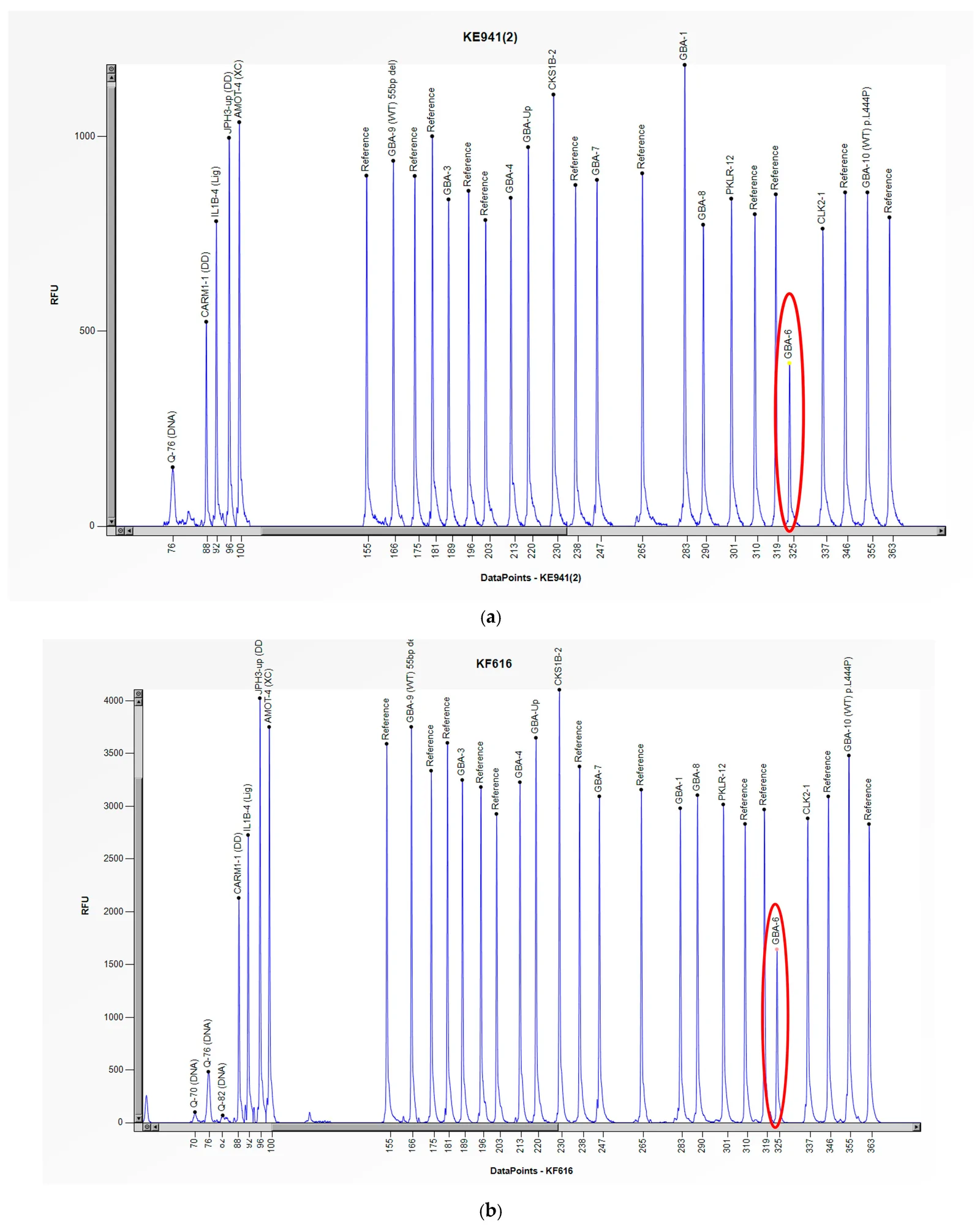
(**a**) The electropherogram of the proband’s sample with code KE941(2) shows a 50% reduction in the peak corresponding to the exon 6 probe of the *GBA-1* gene compared to the reference probe, thus indicating the presence of a heterozygous deletion. (**b**) The electropherogram of the proband’s mother with code KF616 shows a 50% reduction in the peak corresponding to the exon 6 probe of the *GBA-1* gene compared to the reference probe, thus indicating the presence of a heterozygous deletion.

**Figure 6:**
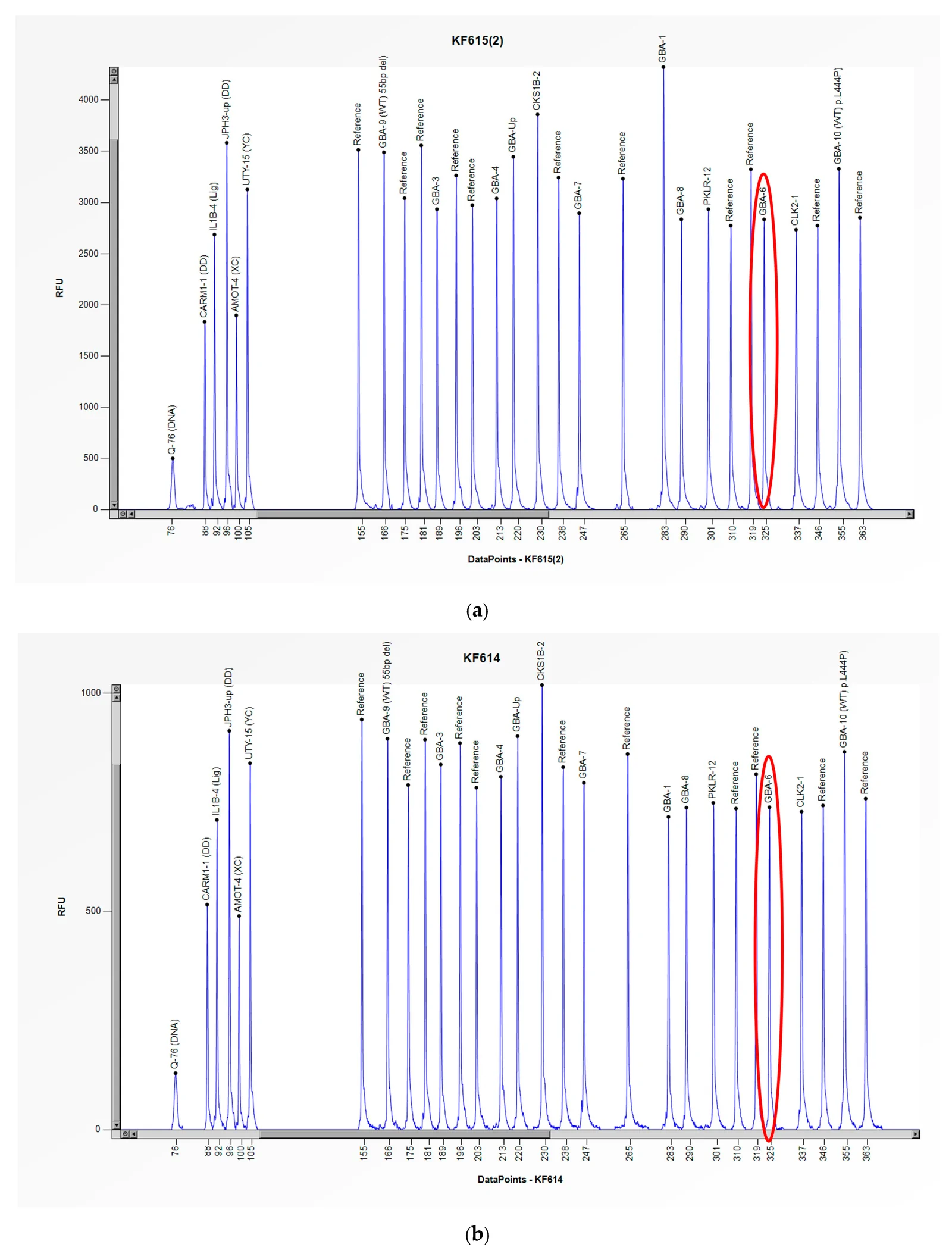
(**a**) The electropherogram of the proband’s father with code KF615(2) shows the standard peak height corresponding to the exon 6 probe of the *GBA-1* gene compared to the reference probe, thus indicating the absence of the deletion. (**b**) The electropherogram of the proband’s brother with code KF614 shows the standard peak height corresponding to the exon 6 probe of the *GBA-1* gene compared to the reference probe, thus indicating the absence of the deletion.

Therefore, MLPA analysis was performed on DNA samples to detect significant rearrangements of the *GBA-1* gene, using the SALSA MLPA P338-B2 GBA kit, in accordance with the manufacturer’s instructions (MRC Holland, Amsterdam, The Netherlands). The kit includes 23 MLPA probes with amplification products ranging from 155 to 363 nucleotides, specific for the *GBA-1* gene; in particular, it includes 9 probes for the *GBA-1* gene and 3 flanking probes, two of which are centromeric and one telomeric. Data analysis was performed using Coffalyser.Net software v.250317.1029. MLPA analysis performed on the proband revealed the presence of a heterozygous deletion of exon 6, manifested by a 50% reduction in the peak height associated with the probe specific for exon 6 of the *GBA-1* gene. The dosage quotient (DQ) for exon 6 was 0.55 (normal values: 0.80 ≤ DQ ≤ 1.20), confirming the presence of the deletion in heterozygosity. This variant was transmitted by the mother, who also had the same heterozygous deletion with a DQ of 0.6 (Figure 5a,b). MLPA analysis performed on the father did not reveal any deletions; in fact, the peak height corresponding to the exon 6 probe was within normal parameters with a DQ of 1 (Figure 6a). Similar results were found in the brother (Figure 6b).

## 3. Discussion

Gaucher disease is a rare lysosomal disorder with autosomal recessive transmission, characterized by high clinical and molecular heterogeneity. This phenotypic variability depends on numerous factors, including the nature of pathogenic variants in the *GBA1* gene, the presence of complex alleles, and possible interference from the highly homologous pseudogene *GBAP*. Consequently, the genotype–phenotype correlation is often non-linear and requires a multimodal diagnostic approach.

In the case described, the young patient presented with clinical symptoms consistent with Gaucher disease and, after Sanger sequencing of the *GBA1* gene, appeared homozygous for the p.Asn409Ser variant. However, the glucosylsphingosine (Lyso-Gb1) value of 498 nmol/L was inconsistent with the biochemical profile typically observed in p.Asn409Ser/p.Asn409Ser patients, suggesting the possible presence of a second genetic alteration not detectable by traditional sequencing.

To clarify this discrepancy between clinical phenotype, biochemical data and preliminary genetic results, a family study was conducted. The father was found to be heterozygous for the p.Asn409Ser variant, which is compatible with the transmission of the mutation to his daughter. However, the mother was found to be wild-type in Sanger sequencing, presenting a picture incompatible with the autosomal recessive mechanism of the disease and suggesting a possible methodological limitation.

Therefore, to investigate the potential involvement of structural rearrangements, an analysis was performed using MLPA, a technique specifically designed for the identification of large deletions and duplications. The analysis revealed a deletion of exon 6 in the mother, transmitted to the patient, defining the real genotype of the proband as compound heterozygous p.Asn409Ser/del exon 6. Specifically, p.Asn409Ser variant was inherited from the father, who is heterozygous for p.Asn409Ser and does not carry the exon 6 deletion, whereas the exon 6 deletion was inherited from the mother, who is heterozygous for the deletion and does not carry p.Asn409Ser variant. Thus, parental segregation strongly supports p.Asn409Ser/exon 6 deletion compound heterozygous genotype, with the two variants inferred to be in *trans*. Consequently, the presumed homozygosity detected by Sanger sequencing appeared to be a technical artefact: the maternal allele carrying the exon 6 deletion is not adequately amplified by the assay used and, consequently, the corresponding exon 9 sequence is not detected. As a result, Sanger sequencing detects only the other allele, of paternal origin, carrying p.Asn409Ser variant, thus leading to a false appearance of homozygosity for p.Asn409Ser.

This case demonstrates the importance of using complementary methods in addition to Sanger sequencing, such as MLPA or next-generation techniques, when genetic results are inconsistent with the phenotypic picture or the expected inheritance pattern. The integration of clinical, biochemical and advanced molecular analysis data is essential for correct genotypic definition, more accurate genotype-phenotype correlation and optimal clinical management of patients with Gaucher disease. This approach is particularly important in the case of the *GBA1* gene, which is characterized by high genetic complexity and heterogeneity. In addition to single-nucleotide variants and small insertions/deletions, *GBA1* can exhibit complex recombinant alleles and structural variants, the identification of which is made particularly difficult by the high sequence homology with the adjacent *GBAP1* pseudogene.

## 4. Conclusions

In conclusion, the diagnosis of Gaucher disease requires a multidimensional analytical approach that systematically integrates biochemical, genetic and family data. Extending molecular testing to family members, in addition to analyzing individual cases, is a crucial step to correctly define allelic segregation and accurately interpret genotype-phenotype correlations, which are essential elements in autosomal recessive diseases characterized by high mutational heterogeneity.

Complete genotypic characterization, obtained through the combined use of methods complementary to conventional sequencing, is a fundamental prerequisite not only for increasing diagnostic accuracy, but also for guiding therapeutic decisions, optimizing genetic counseling and ensuring appropriate clinical management of patients with Gaucher disease.

## Figures and Tables

**Table 1:** Molecular and clinical data of the family of the proband. GCase activity is expressed in nmol/mL/h; normal range is ≥2.5 nmol/mL/h. Lyso-Gb1 in blood is expressed in ng/mL; normal range is ≤6.8 ng/mL.

Patient	Sex	Age	Mutation	GCase	Lyso-Gb1	Clinical Signs
Proband	F	22	p.Asn409Ser/deletion of exon 6	**0.6**	**498.0**	Hepatomegaly, splenomegaly, thrombocytopenia
Father	M	58	p.Asn409Ser/wt	4.2	6.8	None info
Mother	F	53	wt/deletion of exon 6	3.0	4.3	None info
Brother	M	24	wt/wt	7.7	5.1	None info

Bold is used to indicate pathological values.

## Data Availability

The original contributions presented in this study are included in the article. Further inquiries can be directed to the corresponding author.
